# Identification and Characterization of a Garlic Virus E Genome in Garlic (*Allium sativum* L.) Using High-Throughput Sequencing from India

**DOI:** 10.3390/plants11020224

**Published:** 2022-01-15

**Authors:** Malyaj R. Prajapati, Aakansha Manav, Jitender Singh, Pankaj Kumar, Amit Kumar, Ravindra Kumar, Satya Prakash, Virendra Kumar Baranwal

**Affiliations:** 1 College of Biotechnology, Sardar Vallabhbhai Patel University of Agriculture and Technology, Meerut 250110, India; malyajrprajapati@gmail.com (M.R.P.); aakanshasingh721@gmail.com (A.M.); panks.svpuat@gmail.com (P.K.); balyan74@gmail.com (A.K.); kumarrk2000@yahoo.com (R.K.); 2 College of Horticulture, Sardar Vallabhbhai Patel University of Agriculture and Technology, Meerut 250110, India; satyaagro@gmail.com; 3 Division of Plant Pathology, ICAR-Indian Agricultural Research Institute, New Delhi 110012, India; vbaranwal2001@yahoo.com

**Keywords:** garlic, high-throughput sequencing, RT-PCR, phylogenetic analysis

## Abstract

Garlic (*Allium sativum* L.) plants exhibiting mosaics, deformation, and yellow stripes symptoms were identified in Meerut City, Uttar Pradesh, India. To investigate the viruses in the garlic samples, the method of high-throughput sequencing (HTS) was used. Complete genome of the garlic virus E (GarV-E) isolate (NCBI accession No. MW925710) was retrieved. The virus complete genome comprises 8450 nucleotides (nts), excluding the poly (A) tail at the 3′ terminus, with 5′ and 3′ untranslated regions (UTRs) of 99 and 384 nts, respectively, and ORFs encoding replicase with a conserved motif for RNA-dependent RNA polymerase (RdRP), TGB1, TGB2, TGB3, serine-rich protein, coat protein, and nucleic acid binding protein (NABP). The sequence homology shared 83.49–90.40% and 87.48–92.87% with those of GarV-E isolates available in NCBI at the nucleotide and amino acid levels, respectively. Phylogenetic analysis showed a close relationship of this isolate from India (MW925710) with GarV-E isolate YH (AJ292230) from Zhejiang, China. The presence of GarV-E was also confirmed by RT-PCR. The present study is the first report of GarV-E in garlic cultivar Yamuna Safed-3 grown in northern India. However, further studies are needed to confirm its role in symptom development, nationwide distribution, genetic diversity, and potential yield loss to the garlic in India.

## 1. Introduction

Garlic (*Allium sativum* L.; Family: *Amaryllidaceae*) is an aromatic bulbous crop native to central Asia and is consumed worldwide as food in addition to traditional remedies for various diseases [1]. It is highly prone to viral infection, which has adversely reduced bulb weight [2]. Garlic crops are often infected by multiple viruses belonging to several genera that are known as the “garlic virus complex” [2]. Many viruses infecting garlic have been identified in India, including *potyvirus* (onion yellow dwarf virus; OYDV, leek yellow stripe virus; LYSV), *carlavirus* (garlic common latent virus; GarCLV, shallot latent virus; SLV), *tospovirus* (iris yellow spot virus; IYSV), *allexivirus* (garlic virus A; GarV-A, garlic virus B; GarV-B, garlic virus C; GarV-C, garlic virus D; GarV-D and garlic virus X; GarV-X) [3,4,5,6,7,8,9,10,11,12]. Garlic virus E (GarV-E) belongs to the single-stranded, positive-strand RNA virus of the genus *Allexivirirus* and family *Alphaflexiviridae*. It was previously reported in garlic from China, Poland, Australia, the USA, and Japan [13,14,15,16]. These viruses are reported to be transmitted by an insect mite vector [17], vegetative propagation, and mechanically [18]. The disease symptoms include leaf mosaic, deformation, and yellow stripes, which reduce yield and deteriorate the quality of the crop. Because of vegetative propagation, these viruses can accrete in the bulb and can be transmitted to successive generations. Hence, the eradication of these viruses becomes onerous. Considering the importance of garlic, identification, characterization of the virus associated with the disease, and an appropriate management strategy are required [19].

To date, in the public domain, only four complete genomic sequences of GarV-E isolates have been reported from China [20,21]. Several studies have reported genetic differences based on coat protein (CP) sequences within *Allexivirus* species [21], and there are currently 15 partial CP/NABP sequences of GarV-E submitted globally available in the NCBI database [16,22].

In India, the occurrence of GarV-E has not been previously reported. In this present study, we report, for the first time, and characterize the complete genome sequence of GarV-E from garlic in India. Discovering and determining the sequences of more isolates worldwide is important for our understanding of the molecular diversity and evolution of the virus.

## 2. Materials and Methods

### 2.1. Sample Collection, RNA Extraction, High-Throughput Sequencing

In 2018, mild mosaic-like symptoms were observed on the leaves of the aforementioned garlic (*Allium sativum* L.) cultivar Yamuna Safed-3 (G-282) grown at the experimental fields of Horticulture Research Center, Sardar Vallabhbhai Patel University of Agriculture and Technology, Meerut, India. To identify the causative agent(s), total RNA was isolated using TRIzol reagent (BR Biochem Life Sciences, India) from all 16 symptomatic leaf and bulb samples of the garlic cultivar Yamuna Safed 3 (G-282). The amount of extracted RNA was quantified by Qubit (Life Technologies, Carlsbad, CA, USA), and quality was determined using a Bioanalyzer (Agilent Technologies, Santa Clara, CA, USA). Two RNA-Seq libraries were constructed separately using 200 ng of total RNA from the pooled tissues of garlic cloves and leaves from two different garlic plants. These two plants were among the sixteen samples collected from the field. To acquire the plant virus RNA genome from the samples, selective depletion of rRNA using RNaseH (Thermo Scientific, Waltham, MA, USA) was performed. mRNA purification using oligo-dT beads was not executed because it cannot trap viral genomes due to the nonexistence of polyA tails in RNA viruses [23]. The sequence library obtained was allowed for a quality check using a Bioanalyzer (Agilent, Santa Clara, CA, USA). Paired-end 2 × 150 bp and index sequencing were conducted using an Illumina HiSeq 2000 (Illumina Inc., San Diego, CA, USA) at NxGenBio Life Sciences, New Delhi, India.

### 2.2. Virus Identification and Reference Mapping of the Assembled De Novo Contigs

The raw reads obtained were cleaned to remove ambiguous nucleotides, adaptor sequences, and empty reads using CLC Genomic workbench 20.0.4. Taxonomic profiling of Illumina sequencing reads was performed by OmicsBox 1.2 (https://www.biobam.com/omicsbox, accessed on 3 November 2021) using the Kraken 2 database containing WGS RefSeq genomes of archaea, bacteria, fungi, and virus, and the results were visualized by a rich visualization tool in OmicsBox 1.2. De novo assembly was performed to combine sequence reads with overlapping regions to generate longer fragments. These longer reads were allowed for sequence annotation using the BLASTn and BLASTx programs against the Nr database. The alignment against the reference viral genome database was performed using OmicsBox 1.2. Reference-based mapping was also performed with the complete genome sequences of the virus against the most similar existing viral genomes using the read mapping module of the CLC genomics workbench version 20.0.4. The open reading frames (ORFs) encoded by the genome were analyzed by ORF Finder (www.ncbi.nlm.nih.gov/projects/gorf/, accessed on 3 November 2021) to identify the conserved domains present in the virus genome using NCBI’s Conserved Domain-Search tool (https://www.ncbi.nlm.nih.gov/Structure/cdd/wrpsb.cgi, accessed on 3 November 2021), and the genome organization of the virus was constructed using the Bioedit 7.2 program [24].

### 2.3. Pairwise Alignment, Phylogenetic and Recombination Analysis

Alignments of the complete genome sequence of the virus and encoded proteins were made using the ClustalW method in Molecular Evolutionary Genetics Analysis (MEGA) X using default settings [25]. The pairwise sequence identity of viral genomes and encoded proteins was determined using ClustalW in Sequence Demarcation Tool (SDT v1.2) [26]. To construct the phylogenetic trees, selected amino acid and nucleotide alignments were first subjected to model testing in MEGA X [25]. A neighbor-joining model was constructed with strict nucleotide or amino acid distances and a bootstrap value of 1000 replicates using MEGA X [25].

### 2.4. RT-PCR Assay for Detection of GarV-E and Sanger Sequencing

To confirm GarV-E in the collected garlic plants (*n* = 16), including samples used for HTS, degenerate primers spanning the partial CP/NABP gene of *Allexivirus* genus members (Allex-CP (+): (5′-TGGRCXTGCTACCACAAYGG-3′) and Allex-NABP (−): (5′-CCYTTCAGCATATAGCTTAGC-3′) were used in the polymerase chain reaction [21]. cDNA was prepared by reverse transcription using a RevertAid Reverse Transcriptase kit (Thermo Scientific, Waltham, MA, USA) with the degenerate reverse primer of the CP/NABP gene of the *Allexivirus* genus. Two microliters of the resultant cDNA were used to perform PCR in a 25 μL reaction comprising 1 μL of forward (10 µM) and reverse primer (10 µM) and 12.5 μL of Dream Taq PCR Master Mix (Thermo Scientific, Waltham, MA, USA). Thermocycling conditions were set as the initial denaturation step for 10 min at 94 °C, followed by cyclic denaturation at 94 °C for 30 s, primer annealing for 45 s at 58.1 °C, and elongation at 72 °C for 1 min. The reaction was run for 35 cycles with a final elongation step at 72 °C for 10 min. The PCR amplicons were visualized by gel electrophoresis using 0.8% (*w/v*) prestained agarose gel with EtBr (Thermo Scientific, Waltham, MA, USA) and run in 1X TAE buffer. Positive amplicons were purified using the GeneJET PCR Purification Kit (Thermo Scientific, Waltham, MA, USA), cloned in the pGEM-T vector (Promega, Madison, WI, USA), and sequenced bidirectionally by Sanger sequencing (Applied Biosystems, Beverly, MA, USA) at Biokart India Pvt. Ltd. (Bengaluru, India).

## 3. Results and Discussion

### 3.1. Sequence Analysis

To reveal viruses that might be associated with the symptoms, the RNA of pooled symptomatic clove and leaf samples was sequenced using the Illumina HiSeq 2000 platform. The size of the Illumina sequencing data generated was approximately 43 million 125 bp paired-end reads in the two libraries. After trimming 34,873,264 bp (average length 124.54 bp) and 31,494,032 bp (average length 124.57 bp), raw sequence reads were obtained (Table S1). A total of 133,971 and 108,668 contigs were generated from clove and leaf samples of garlic, respectively. All contigs were subjected to a BLASTn search against the nr database, which revealed whole-genome sequences of GarV-E apart from other garlic viruses, including *potyvirus* (onion yellow dwarf virus; OYDV, leek yellow stripe virus; LYSV), *carlavirus* (garlic common latent virus; GarCLV, shallot latent virus; SLV), and *allexivirus* (garlic virus A; GarV-A, garlic virus B; GarV-B, garlic virus C; GarV-C, garlic virus D; GarV-D and garlic virus X; GarV-X). Sequence taxonomic profiling was visualized using a Krona graph, and sequence reads belonging to *Allexivirus*, GarV-A (14%), GarV-B (5%), GarV-D (36%), GarV-E (18%), and GarV-X (27%), were obtained (Figure S1). The sequence mapping, BLAST analysis, and Kraken approach generated the complementary datasets, which were supported with 100% convergence. Reference-based mapping of the data revealed that the viral reads mapped to GarV-A, GarV-D, GarV-E, GarV-X, OYDV, LYSV, and GarCLV in both of the samples (Table S2). The obtained data revealed that 100,184 (0.33%) reads from clove and 510,948 (1.95%) reads from leaf sample mapped with the GarV-E genome.

### 3.2. Genome Annotation and Analysis of Garlic Virus E

**Figure 1:**
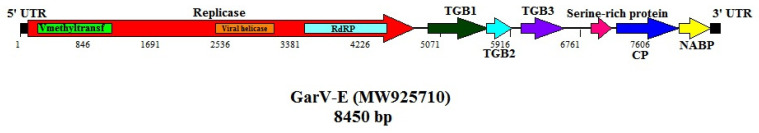
Genomic organization of GarV-E (MW925710) showing seven predicted open reading frames and their corresponding products: replicase, TGB1, TGB2, TGB3, serine-rich protein, viral coat protein (CP), and nucleic acid binding protein (NABP).

BLASTn program-based analysis showed that the GarV-E contig comprises the complete genome sequence of 8450 bp ssRNA. The 5′ UTR and 3′ UTR sequences were not included in the study, and the genome sequence obtained in the study was deposited to NCBI with accession number MW925710. In addition to exploring the amino acid sequence in all possible open reading frames (ORFs), it was viable to detect the characteristic domains along with conserved motifs specific to the genus *Allexivirus* [13,27,28,29]. The ORF Finder and smart BLAST tool revealed that ORF1 encodes replicase (4671 nt; 1556 aa) with a conserved motif SG×3T×3NT×22GDD, which is the proposed active site of the RNA-dependent RNA polymerase (RdRP), was found at amino acid positions 1317–1353, ORF2 a TGB1 (735 nt; 244 aa), ORF3 a TGB2 (309 nt; 102 aa), ORF4 a TGB3 (225 nt; 74 aa), ORF5 a serine-rich protein (234 nt; 77 aa), ORF6 a coat protein (759 nt; 252 aa), and ORF7 an NABP (348; 127 residues) (Figure 1). The pairwise sequence comparison at the level of nucleotides and deduced amino acids of all seven ORFs revealed 72.8–98.3% and 80.5–98.7% identities, respectively, with the Chinese isolates (AJ292230, MN059326, MN059327, and MN059328) (Table 1).

### 3.3. Sequence Similarity and Phylogenetic Analysis

BLASTn [30] searches of the NCBI databases showed that the complete genome of GarV-E isolate India (MW925710) shared 83.49–90.40% nucleotide sequence identities with previously reported isolates (AJ292230, MN059326, MN059327, and MN059328). Moreover, the Indian isolate was more closely related to isolate YH (AJ292230) (90.40%) from Zhejiang, China. A similar result was obtained using an NJ-based phylogenetic tree of the complete genome sequence of GarV-E with other complete genome sequences of GarV-E and other *Allexivirus* species from different regions of the world.

**Figure 2:**
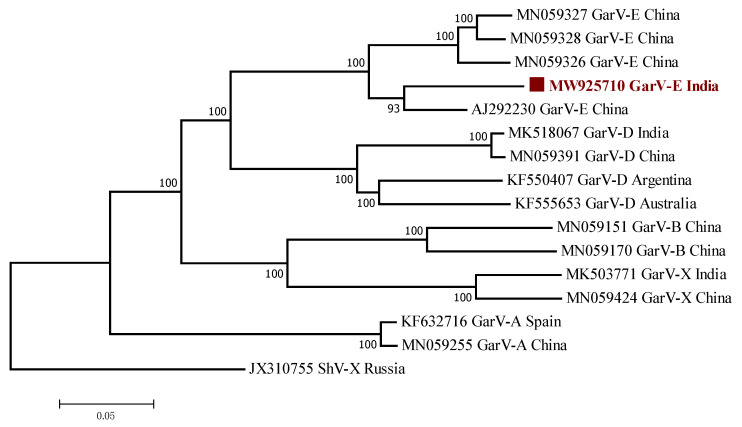
Phylogenetic analysis of GarV-E isolates in the complete genome amino acid sequence using Neighbor joining algorithm. The evolutionary distances were computed using p-distance method with 1000 bootstrap replicates. The scale bar indicates the number of substitutions per site.

In this study, ingroups were selected from the same species from different countries based on the closely related complete genome, and complete CP sequences to the respective viruses and outgroups were selected from the *Allexivirus* genus virus containing enough homologous sites to the respective ingroup virus species to assess the evolutionary relationship. The phylogenetic tree revealed that GarV-E Indian isolates (accession no. MW925710) grouped in the same clade as other GarV-E isolates reported from other countries (Figure 2). Similar phylogenetic tree results were obtained at amino acid (aa) level. The pairwise sequence identities (%) of the GarV-E complete genome sequence (MW925710) shared nucleotide (nt) identity at 79.80–90.10% and amino acid (aa) identity at 79.90–89.1% with other GarV-E isolates reported globally (Table 2).

The results of the phylogenetic tree constructed with nucleotide sequences of the complete coat protein (CP) gene available in NCBI for the genus *Allexivirus* were consistent with the results obtained for the complete genome. Previously, in many of the assessments between members of diverse species of *Allexivirus*, the percent nt and aa sequence identities of the CP gene showed values greater than those suggested by ICTV. This was also identified by [21], who proposed that GarV-A may be combined with other viruses, such as GarV-D and GarV-E, providing more than 73% nt identity among the CP genes to become acceptable for GarV species characterization. Similarly, GarV-B may be combined with GarV-X [21]. The comparison of the CP gene of GarV-E Meerut India (MW925710) with similar sequences shared nucleotide (nt) identity at 83.3–90.6% and amino acid (aa) identity at 86.5–92.8% with other GarV-E isolates reported globally (Table 1). 

Out of 16 samples, seven, including samples used for HTS, were found to be positive for virus infection using RT-PCR with an amplicon of ~750 bp. The sequences obtained were deposited to NCBI with accession numbers MW925695, OK064618, OK064619, OK064620, and OK064621. BLASTn analysis of the partial CP/NABP gene of GarV-E India (MW925695) shared 83.63–92.96% nucleotide identity with other isolates available in NCBI. The pairwise sequence identity comparison of the partial CP/NABP gene of GarV-E India (MW925695) with similar sequences shared nucleotide (nt) identity at 83.5–92.9% and amino acid (aa) at 82.5–96.7% (Table S3). Moreover, the Indian isolate shared a high sequence identity with E-JF-2 isolate (LC097189) (90.40% nt and 96.7% aa) isolated from Fukuoka, Japan. To better understand the genetic variability of the GarV-E isolates, we selected 18 partial CP/NABP coding region sequences of GarV-E and other *Allexivirus* from different geographical locations to construct a phylogenetic tree. In the phylogenetic tree, the GarV-E partial CP/NABP India isolate (accession no. MW925695) was grouped in the same clade as other GarV-E isolates reported from other countries (Figure S2).

NJ-based phylogenetic analysis of the complete genome of the virus (Figure 2), complete coat protein region, and partial CP/NABP coding region (Figure S2) showed consistent clustering of isolates. They all suggest a close relationship between the Indian GarV-E isolate and other GarV-E isolates, supported by high posterior probability values. Recombination appears to be rare in single-stranded, negative-sense RNA viruses, although for those with segmented genomes, such as influenza A, a genetic exchange can still occur through reassortment [31]. We did not find any strong signatures of recombination by RDP4 in individual alignments of the Indian GarV-E isolate (data not presented).

## 4. Conclusions

Viruses are major constraints on global garlic production, including India. Along with expanding global trade, the chance of migrating garlic viruses is mounting daily. Consequently, rapid and reliable garlic virus detection techniques are mandatory to stop this migration as well as for virus-free seed garlic production. In this study, we used HTS to determine the complete genome sequence of GarV-E. This was performed using a dual strategy involving direct mapping of reads against a collection of reference sequences of garlic infecting viruses by de novo assembly of reads, followed by BLASTn/BLASTx annotation of contigs. We also describe the first report of GarV-E from the garlic cultivar Yamuna Safed-3 grown in northern India. A comparative analysis of the genome sequences of additional GarV-E isolates would be helpful to give a clearer picture of genetic variability and evolution of this important virus. Such a study may also play an important role in developing GarV-E disease management strategies. However, biological studies are needed to identify mechanisms of transmission and to assess the effects of single/mixed infection on garlic plants. Disease-free plant of garlic (*Allium sativum* L.) can be produced by plant tissue culture techniques, but they infect again in the open field.

## Figures and Tables

**Table 1:** Pairwise percent sequence identity of Indian GarV-E Isolate (MW925710) at the nucleotide (nts) level and its deduced amino acid (aa) sequence of ORFs with other complete genomes of *Allexiviruses* (for which complete genome sequences are available).

	Replicase	TGB 1	TGB 2	TGB3	Serine-Rich Protein	CP	NABP
AJ292230_GarV-E_China	90.2 (92.87)	86.9 (89.9)	90.7 (92.2)	85.7 (88.2)	88.4 (88.3)	90.6 (92.8)	93.4 (95.1)
MN059326_GarV-E_China	82.8 (87.48)	98.3 (98.7)	84.9 (84.4)	72.8 (80.5)	77.7 (81.8)	84.0 (87.6)	86.9 (90.5)
MN059327_GarV-E_China	82.5 (87.60)	94.9 (97.5)	86.5 (86.4)	72.8 (80.5)	78.2 (81.8)	83.1 (86.5)	86.1 (89.7)
MN059328_GarV-E_China	82.5 (87.54)	94.6 (97.5)	86.8 (83.4)	75.3 (81.2)	78.6 (83.1)	83.3 (87.3)	85.3 (87.1)
MK518067_GarV-D_India	80.6 (82.09)	57.3 (61.3)	76.6 (82.5)	66.6 (72.8)	75.2 (72.7)	74.8 (77.1)	71.8 (74.9)
MN059391_GarV-D_China	79.8 (81.40)	57.7 (61.3)	76.6 (82.5)	66.2 (72.8)	75.2 (72.7)	74.9 (77.1)	73.4 (74.3)
KF550407_GarV-D_Argentina	76.7 (80.6)	57.3 (60.0)	73.7 (79.6)	61.7 (66.2)	69.2 (68.8)	71.1 (75.5)	72.8 (74.6)
KF555653_GarV-D_Australia	74.8 (77.9)	58.1 (60.3)	73.7 (80.5)	57.7 (61.4)	68.3 (68.8)	70.7 (76.7)	73.8 (74.1)
KF632716_GarV-A_Spain	59.0 (61.5)	56.1 (57.6)	73.0 (77.6)	61.7 (66.8)	67.9 (64.9)	67.4 (72)	69.3 (74.5)
MN059255_GarV-A_China	58.9 (62.7)	56.3 (57.6)	73.0 (77.6)	61.7 (65.5)	66.6 (63.6)	67.3 (71.6)	68.8 (73.7)
MN059151_GarV-B_China	65.8 (68.3)	53.1 (52.8)	58.9 (48.0)	37.1 (47.5)	54.2 (57.1)	54.2 (57.3)	63.8 (70.6)
MK503771_GarV-X_India	65.4 (67.9)	53.2 (55.9)	56.0 (53.8)	40.0 (51.0)	50.4 (49.3)	56.2 (56.9)	64.4 (69.0)
MN059424_GarV-X_China	65.7 (68.2)	53.2 (55.1)	57.6 (51.9)	39.6 (43.5)	51.7 (51.9)	57.3 (57.3)	64.9 (74.7)
MN059170_GarV-B_China	66.0 (68.2)	52.4 (52.6)	58.6 (49.0)	39.3 (43.0)	53.8 (49.3)	55.8 (57.6)	56.3 (62.8)
JX310755_ShV-X_Russia	55.7 (58.5)	60.7 (63.6)	61.5 (58.2)	41.6 (44.1)	58.1 (55.8)	57.7 (60.0)	68.8 (72.4)

**Table 2:** Comparisons of nucleotide sequence (nts) and amino acid (aa) identity of pairwise combinations of complete genome sequences of garlic virus E (accession no. MW925710) with other complete genome sequences of *Allexiviruses*. (for which complete genome sequences are available).

Seq-> nts/aa	MW925710_GarV-E_India	AJ292230_GarV-E_China	MN059326_GarV-E_China	MN059327_GarV-E_China	MN059328_GarV-E_China	MK518067_GarV-D_India	MN059391_GarV-D_China	KF550407_GarV-D_Argentina	KF555653_GarV-D_Australia	KF632716_GarV-A_Spain	MN059255_GarV-A_China	MN059151_GarV-B_China	MN059170_GarV-B_China	MK503771_GarV-X_India	MN059424_GarV-X_China	JX310755_ShV-X_Russia
MW925710_GarV-E_India	ID	89.1	80.1	79.9	82.9	71.6	71.6	70.4	70.3	57.4	57.1	59.6	60.2	59.5	60.1	52.7
AJ292230_GarV-E_China	90.1	ID	84.9	84.9	81.2	61.7	61.8	61.2	62.1	52.4	52.2	52.5	52.7	52.5	53.0	47.9
MN059326_GarV-E_China	83.7	87.9	ID	95.6	91	75	75.1	74.4	75	63	62.9	64.3	64.3	63.9	64.6	57.7
MN059327_GarV-E_China	83.1	88.1	94.5	ID	91.7	74.6	74.7	74.4	75.3	63	62.9	64.2	64.2	64.1	64.8	57.8
MN059328_GarV-E_China	79.8	83.6	89.7	91.5	ID	91.7	74.6	74.7	74.4	75.3	63	62.9	64.2	64.2	64.1	64.8
MK518067_GarV-D_India	75.1	68.9	68.7	68.3	65.2	ID	99.6	90.4	89.6	63.6	62.9	63.4	63.7	62.9	63.6	57.4
MN059391_GarV-D_China	74.6	68.8	68.7	68.2	65.1	98.6	ID	90.3	89.5	63.8	63	63.5	63.8	63	63.7	57.5
KF550407_GarV-D_Argentina	71.8	69.3	69.2	69.1	66.3	85.4	84.5	ID	89.2	63.8	63	63.2	63.5	63.1	63.7	58
KF555653_GarV-D_Australia	75	69.4	69.0	68.8	65.8	83.2	83.7	86.4	ID	63.4	62.8	63.5	63.5	62.8	63.3	57.9
KF632716_GarV-A_Spain	65	61.7	61.3	61.5	58.7	61.7	61.5	62.1	6.2	ID	78.7	57.3	57.5	57.3	57.8	64.4
MN059255_GarV-A_China	65	61.6	61.2	61.4	58.6	61.6	61.4	62.0	61.8	98.3	ID	56.9	56.9	56.6	57	63.1
MN059151_GarV-B_China	59.8	61.1	61.2	61.1	59.1	61.0	61.1	69	61.1	57.3	57.3	ID	90.8	75.8	75.2	54.2
MN059170_GarV-B_China	59.7	61.0	61.1	61.2	59.2	65	66	66	67	56.9	57.1	86.4	ID	74.7	75.3	54
MK503771_GarV-X_India	59.6	67	68	67	58.8	61.2	61.3	61.3	61.0	56.4	56.5	77	70	ID	90.6	54.3
MN059424_GarV-X_China	59.9	61.1	69	68	59.0	67	61.0	68	61.2	56.7	56.8	73	69.9	99	ID	54.3
JX310755_ShV-X_Russia	55.7	56.5	56.3	56.6	54.1	56.7	56.8	56.4	56.4	63.4	63.5	54.3	54.1	54.1	54.2	ID

## Data Availability

The complete nucleotide sequences of garlic virus E (GarV-E) have been deposited in NCBI under accession number MW925710, and partial CP/NABP gene sequences have been deposited under accession numbers MW925695, OK064618, OK064619, OK064620, and OK064621.

## Supplementary Materials

The following supporting information can be downloaded at: https://www.mdpi.com/article/10.3390/plants11020224/s1. Table S1: HTS statistics for each sample before and after quality control showing the number of reads and the range of read lengths in base pairs (bp). Figure S1. Krona plot of *Allexivirus* genome sequence reads generated on Illumina sequencing platforms. Table S2: Number of viral reads mapped to a reference sequence. Table S3: Comparisons of nucleotide sequence (nts) and amino acid (aa) identity of pairwise combinations of partial CP/NABP sequences of garlic virus E (accession no. MW925695) with other isolates of garlic viruses infecting garlic reported worldwide. Figure S2: Phylogenetic analysis of GarV-E isolates in the partial CP/NABP genome sequence using neighbor joining algorithm. The evolutionary distances were computed using p-distance method with 1000 bootstrap replicates. The scale bar indicates the number of substitutions per site.
