# Supplementary material for: Identification and Characterization of a Garlic Virus E Genome in Garlic (Allium sativum L.) Using High-Throughput Sequencing from India
Source: Plants (Basel). 2022 Jan 15;11(2):224. doi: 10.3390/plants11020224 (PMC8780593; doi:10.3390/plants11020224)
Supplement: Supplementary file 1 [file plants-11-00224-s001.zip › plants-1502533-supplementary.pdf]

plants MDPI

Table S1. HTS statistics for each sample before and after quality control showing the number of reads and the range of read lengths in base pairs (bp).

Sample	Input Reads	Average length	No. of Read after Trimming	Percentage Trimmed (%)	Average Length after Trim
Clove	34,873,376	125.0	34,873,264	100.0	124.54
Leaf	31,497,56	124.99	31,494,032	99.99	124.57

Table S2. Number of viral reads mapped to a reference sequence.

Sample	Total reads	GarV-E	GarV-A	GarV-D	GarV-X	LYSV	OYDV	GarCLV
Clove	34,873,152	100,184 **(0.33%)**	144,529 **(0.41%)**	239,254 **(0.69%)**	97,926 **(0.28%)**	510,948 **(1.95%)**	4,817 **(0.01%)**	144,529 **(0.41%)**
Leaf	31,49,974	510,948 **(1.95%)**	1,004,995 **(3.19%)**	100,184 **(0.33%)**	265,986 **(0.84%)**	25,009 **(0.08%)**	21,121 **(0.07%)**	1,004,995 **(3.19%)**

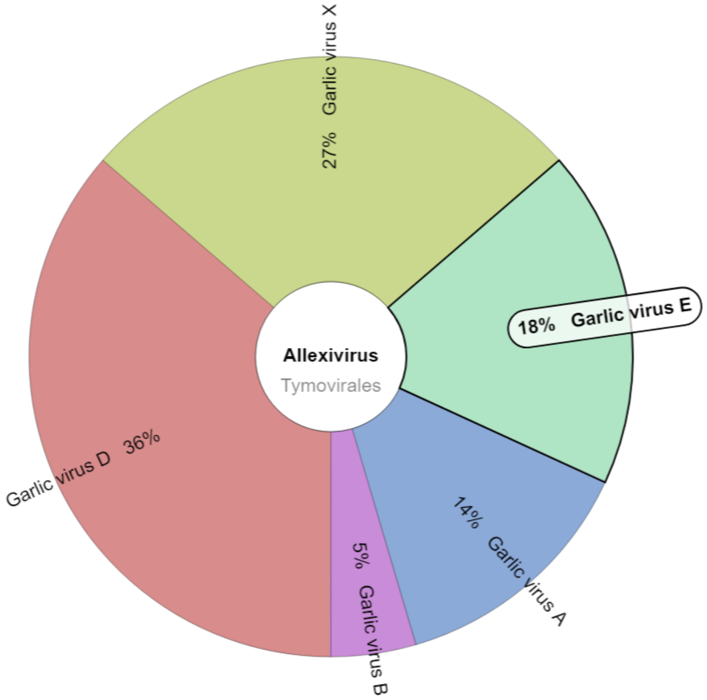

Figure S1. Krona plot of Allexivirus genome sequence reads generated on Illumina sequencing platforms.

Table S3. Comparisons of nucleotide sequence (nts) and amino acid (**aa**) identity of pairwise combinations of partial CP/NABP sequences of garlic virus E (accession no. MW925695) with other isolates of garlic viruses infecting garlic reported worldwide.

**Seq->** nts/**aa**	**MW925695_GarV-E_India**	**LC097189_GarV-E_Japan**	**LC097188_GarV-E_Japan**	**LC097187_GarV-E_China**	**LC097186_GarV-E_USA**	**LC097185_GarV-E_USA**	**LC097184_GarV-E_USA**	**AJ551502_GarV-E_China**	**AJ551501_GarV-E_China**	**AJ551500_GarV-E_China**	**AJ551499_GarV-E_China**	**AJ551498_GarV-E_China**
MW925695_GarV-E_India	ID	**96.7**	**96.3**	**96.3**	**95.9**	**96.3**	**96.3**	**84.5**	**84.9**	**84.1**	**85.3**	**82.5**
LC097189_GarV-E_Japan	92.9	ID	**99.5**	**99.5**	**99.1**	**99.5**	**99.5**	**86.9**	**87.3**	**86.5**	**87.8**	**84.1**
LC097188_GarV-E_Japan	92.5	99.5	ID	**100**	**99.5**	**100**	**100**	**86.9**	**87.3**	**86.5**	**87.3**	**83.7**
LC097187_GarV-E_China	92.5	99.5	99.8	ID	**99.5**	**100**	**100**	**86.9**	**87.3**	**86.5**	**87.3**	**83.7**
LC097186_GarV-E_USA	92.3	99.4	99.8	99.7	ID	**99.5**	**99.5**	**86.9**	**87.3**	**86.5**	**87.3**	**83.3**
LC097185_GarV-E_USA	92.6	99.7	99.8	99.8	99.7	ID	**100**	**86.9**	**87.3**	**86.5**	**87.3**	**83.7**
LC097184_GarV-E_USA	92.6	99.7	99.8	99.8	99.7	100	ID	**86.9**	**87.3**	**86.5**	**87.3**	**83.7**
AJ551502_GarV-E_China	86.1	89.6	89.4	89.4	89.3	89.6	89.6	ID	**99.5**	**99.1**	**85.2**	**83.9**
AJ551501_GarV-E_China	86.3	89.7	89.8	89.7	89.7	89.7	89.7	98.2	ID	**98.7**	**85.6**	**84.3**
AJ551500_GarV-E_China	86.1	89.4	89.6	89.6	89.4	89.7	89.7	98.6	98.2	ID	**84.8**	**83.5**
AJ551499_GarV-E_China	85.2	88.9	88.8	88.8	88.6	88.9	88.9	88.0	88.5	88.0	ID	**88.0**
AJ551498_GarV-E_China	83.5	86.6	86.4	86.4	86.3	86.6	86.6	86.6	86.8	86.4	88.4	ID

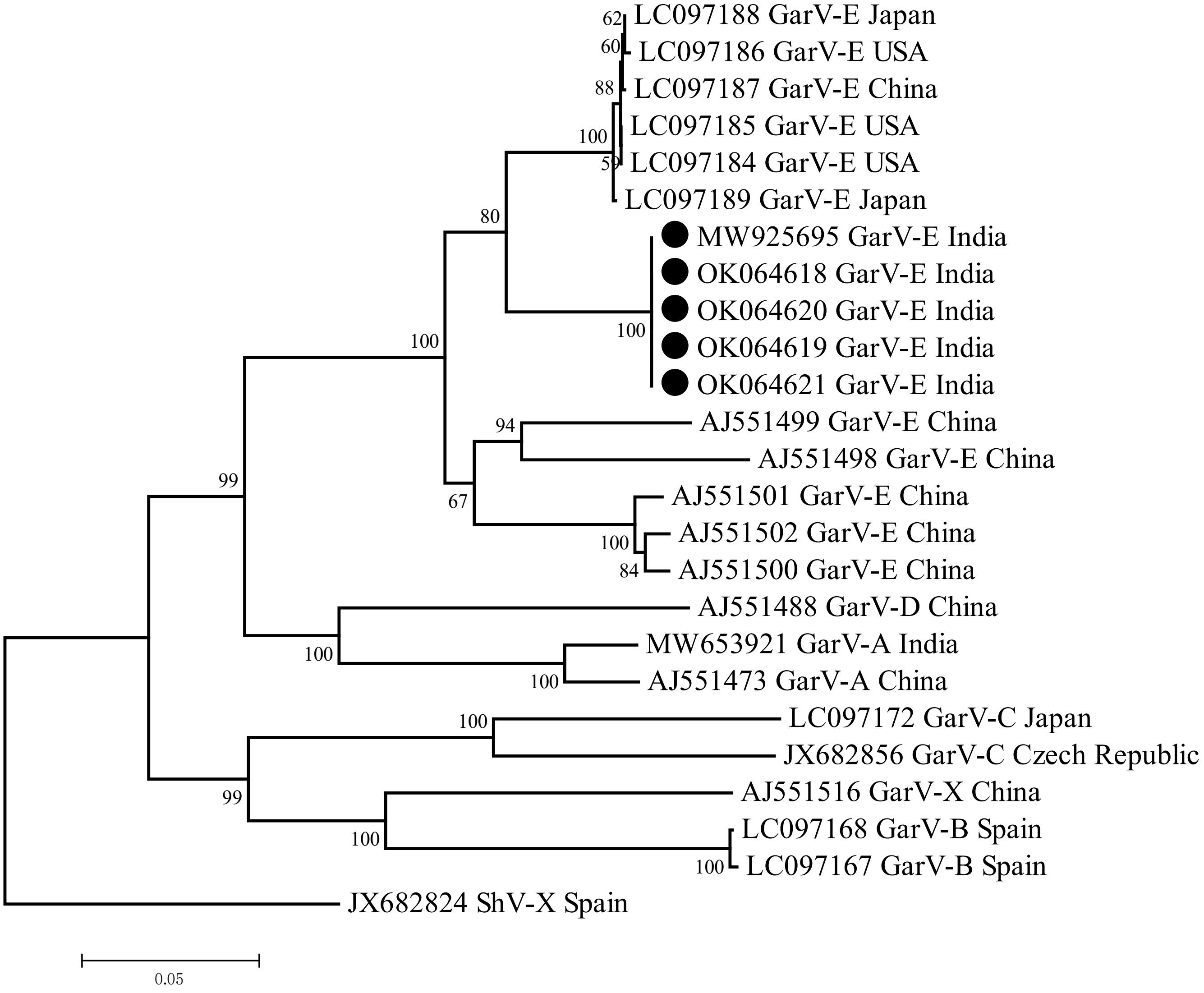

Figure S2. Phylogenetic analysis of GarV-E isolates in the partial CP/NABP genome sequence using Neighbour joining algorithm. The evolutionary distances were computed using p-distance method with 1000 bootstrap replicates. The scale bar indicates the number of substitutions per site.
